# Anthracycline-induced cardiotoxicity and senescence

**DOI:** 10.3389/fragi.2022.1058435

**Published:** 2022-11-14

**Authors:** Laura K. Booth, Rachael E. Redgrave, Omowumi Folaranmi, Jason H. Gill, Gavin D. Richardson

**Affiliations:** ^1^ School of Pharmacy, Translational and Clinical Research Institute, Vascular Biology and Medicine Theme, Newcastle University, Newcastle upon Tyne, United Kingdom; ^2^ Biosciences Institute, Vascular Biology and Medicine Theme, Newcastle University, Newcastle upon Tyne, United Kingdom

**Keywords:** chemotherapy, anthracyclines, cancer, cardiac, heart failure, senescence, senolytic

## Abstract

Cancer continues to place a heavy burden on healthcare systems around the world. Although cancer survivorship continues to improve, cardiotoxicity leading to cardiomyopathy and heart failure as a consequence of cancer therapy is rising, and yesterday’s cancer survivors are fast becoming today’s heart failure patients. Although the mechanisms driving cardiotoxicity are complex, cellular senescence is gaining attention as a major contributor to chemotherapy-induced cardiotoxicity and, therefore, may also represent a novel therapeutic target to prevent this disease. Cellular senescence is a well-recognized response to clinical doses of chemotherapies, including anthracyclines, and is defined by cell cycle exit, phenotypic alterations which include mitochondrial dysfunction, and the expression of the pro-senescent, pro-fibrotic, and pro-inflammatory senescence-associated phenotype. Senescence has an established involvement in promoting myocardial remodeling during aging, and studies have demonstrated that the elimination of senescence can attenuate the pathophysiology of several cardiovascular diseases. Most recently, pharmacology-mediated elimination of senescence, using a class of drugs termed senolytics, has been demonstrated to prevent myocardial dysfunction in preclinical models of chemotherapy-induced cardiotoxicity. In this review, we will discuss the evidence that anthracycline-induced senescence causes the long-term cardiotoxicity of anticancer chemotherapies, consider how the senescent phenotype may promote myocardial dysfunction, and examine the exciting possibility that targeting senescence may prove a therapeutic strategy to prevent or even reverse chemotherapy-induced cardiac dysfunction.

## Introduction

Cancer continues to place a heavy burden on healthcare systems around the world. In line with the aging and growing population (Smittenaar et al., 2016), it is now estimated that the lifetime risk of cancer for people born after the 1960s is greater than 50%, notably larger than the same estimate for those born in the 1930s (15% increase for men and 10.8% increase for women) (Ahmad et al., 2015). Although cancer survivorship continues to improve, cardiotoxicity, as a consequence of anticancer therapy, is becoming a major concern, which can culminate in cardiomyopathy and heart failure. Consequently, yesterday’s cancer survivors are fast becoming today’s heart failure patients. Indeed, some cancer patients are more likely to die as a result of cardiovascular disease (CVD) than cancer itself, as demonstrated by the observations that CVD is the leading cause of death in female breast cancer patients, and individuals in remission from childhood cancers are more likely to die from CVD than suffer cancer relapse (McGowan et al., 2017). As such, chemotherapy-induced cardiotoxicity is creating a new set of healthcare challenges. Despite substantial evidence that anthracyclines (including the most well-studied anthracycline family member doxorubicin, DOX) can cause heart failure and the development of several new-generation targeted anticancer therapies, anthracyclines remain a prominent clinical tool for cancer treatment (De Angelis et al., 2016) and are the mainstay class of chemotherapeutics against a diverse range of malignancies (Yousefzadeh et al., 2021). Anthracycline-induced cardiotoxicity (AIC) leads to ventricular dysfunction in up to 37.5% of patients receiving chemotherapy (López-Sendón et al., 2020). AIC can manifest as acute toxicity, presenting as arrhythmias and elevated brain natriuretic peptide and troponin levels; however, these effects are usually reversible after therapy discontinuation (Nair and Gongora, 2016). More commonly and clinically important, AIC can also present as late-onset chronic toxicity associated with the irreversible progression to heart failure in the years or even decades after the conclusion of therapy (Von Hoff et al., 1977; Nair and Gongora, 2016). Late-onset AIC presents as pathological myocardial remodeling typified by cardiomyocyte hypertrophy and increased fibrosis leading to functional cardiac decline, which occurs in up to 65% of patients treated with anthracyclines for childhood malignancies and 12% of patients treated with anthracyclines for breast cancer (Grenier and Lipshultz, 1998; Volkova and Russell, 2011). Clinically, dexrazoxane has been shown to have some modest cardioprotective potential; however, not all patient groups respond equally to dexrazoxane, with girls responding to treatment better than boys (Lipshultz et al., 2010). It is also a concern that dexrazoxane may diminish the antineoplastic effects of anthracycline treatment, and although studies up to now have generally been reassuring, this remains a controversial topic in pediatric oncology (Lipshultz et al., 2010; van Dalen et al., 2011).

Ultimately for patients suffering from end-stage heart failure as a result of AIC, heart transplantation remains the only effective treatment (Stehlik et al., 2011; Oliveira et al., 2014). However, patients with AIC are often ineligible for transplant because of the current or previous history of malignancy (Christiansen, 2010) and due to concerns of cancer recurrence in the setting of immunosuppressive therapy (Oliveira et al., 2014). As such, even this last-resort treatment is unavailable to many patients.

It is presumed that late-onset cardiotoxicity is caused by acute subclinical perturbations to cardiac cellular biology which remains asymptomatic and ‘hidden,’ but over months and years, it consequently causes responsive pathological myocardial remodeling, progressing toward symptomatic and fulminant heart failure (Zhao and Zhang, 2017). A possible cell fate that could arise during chemotherapy and contribute to the chronic/progressive nature of AIC (and the associated increased prevalence in CVD) is cellular senescence (Abdelgawad et al., 2021). In this mini-review, we will provide a concise discussion of the evidence that anthracyclines can induce myocardial senescence, consider how the senescent phenotype may promote myocardial dysfunction, and examine the exciting possibility that targeting senescence may prove a therapeutic strategy to prevent or even reverse AIC.

## Chemotherapy and cellular senescence

Cellular senescence is a cellular fate induced by a persistent DNA damage response, which is defined by cell cycle exit and phenotypic alterations, including mitochondrial dysfunction and the expression of the pro-senescent, pro-fibrotic, and pro-inflammatory senescence-associated secretory phenotype (SASP) (Campisi and d'Adda di Fagagna, 2007). Senescence is a hallmark of aging, and an accumulation of senescent cells is observed in several age-related diseases and different tissues during aging (Muñoz-Espín and Serrano, 2014). Since clearance of senescent cells can ameliorate several age-related pathologies in different disease models and increase life and health span, it has been hypothesized that senescence causes aging and age-related pathophysiology (Baker et al., 2011; Baker et al., 2016).

**FIGURE 1:**
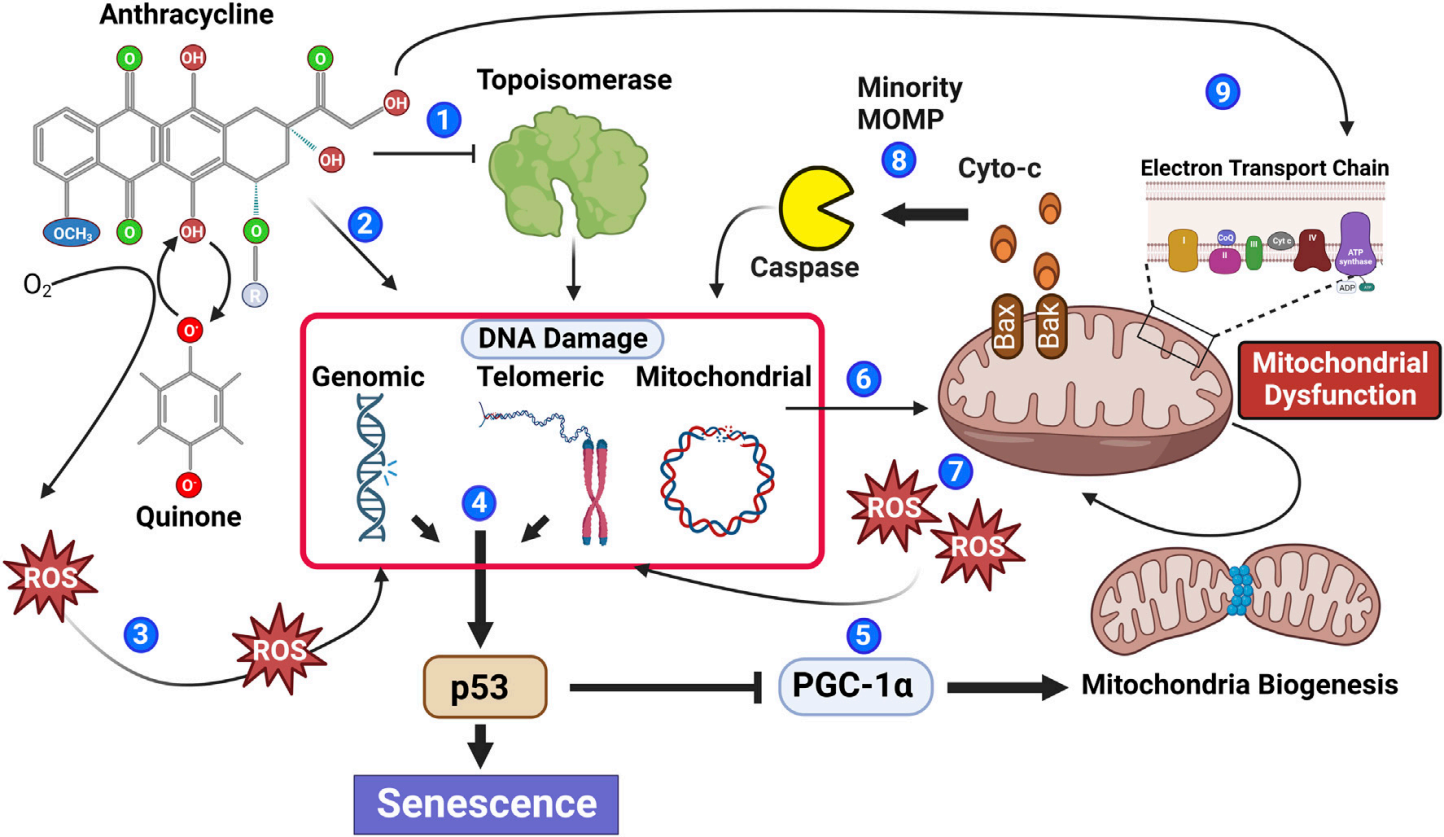
Anthracyclines contribute to multiple biological processes that stimulate senescence. Anthracyclines can inhibit the enzymatic function of topoisomerase leading to irreversible DNA damage (1) and through DNA intercalation induce DNA damage independent of topoisomerase (2). Moreover, the quinone structure of anthracyclines can be oxidized to a semiquinone radical through the addition of an electron. Semiquinone radicals quickly react with oxygen to generate ROS causing DNA damage (3). DNA damage within the genomic or telomeric chromosomal regions leads to activation of the DNA damage response pathway, which if persistent contributes to phosphorylation of the key senescence regulator p53 (4). Phosphorylated p53 inhibits PGC-1a attenuating mitochondrial biogenesis (5). Reduced mitochondrial biogenesis in combination with anthracycline-mediated mitochondrial DNA damage (6) leads to an accumulation of mitochondrial dysfunction and increased ROS generation, contributing to additional DNA damage (7). Mitochondrial outer membrane permeability (MOMP) is also increased in dysfunctional mitochondria allowing the release of caspase-activating cytochrome C; if insufficient to induce apoptosis, the sub-lethal activation of caspase will cause further DNA damage (8). Recently, it has also been suggested that anthracyclines can cause biogenetic lesions to the electron transport chain (Tarpey et al., 2019), further contributing to increased ROS (9).

Anthracyclines including DOX can induce most, if not all, cell types to a senescent phenotype, and therefore, DOX treatment is one of the most utilized tools to study senescence *in vitro* and *in vivo* (Yang et al., 2012; Bielak-Zmijewska et al., 2014; Sun et al., 2022). With regards to the heart, preclinical models have demonstrated that DOX can induce myocardial cell lineages including fibroblasts, endothelial cells, and cardiomyocytes to senescence (Wolf and Baynes, 2006; Maejima et al., 2008; Zhang et al., 2009; Fourie et al., 2019), and *in vivo*, DOX has been shown to induce biomarkers of senescence in the myocardium of mice (Mitry et al., 2020). Senescence induction is a well-recognized response to clinical doses of chemotherapy, indicating a potential contribution for senescence and the SASP in the pathophysiology of clinical AIC (Ewald et al., 2010; Saleh et al., 2020; Wang et al., 2020; Wyld et al., 2020). Furthermore, cancer survivors not only develop CVDs usually associated with increased age but also demonstrate systemic aging-related pathologies such as peripheral neuropathy, a decline in bone health, and cognitive decline which are all consistent with an increase in systemic senescence (Cupit-Link et al., 2017). Although the mechanisms by which anthracyclines induce myocardial cells to senescence are not fully understood (Maejima et al., 2008), for decades, it has been known that anthracyclines damage DNA through intercalation and topoisomerase II “poisoning,” induce mitochondrial dysfunction (thereby promoting reactive oxygen species generation and increasing oxidative stress) (Zhang et al., 2012), and trigger caspase activation, all of which have been implicated in senescence induction (Figure 1) and reviewed elsewhere (Anderson et al., 2018; Owens et al., 2021; Dookun et al., 2022). Providing compelling evidence that senescence contributes to AIC are the studies of Demaria and colleagues. Using a transgenic model that allows the specific identification and elimination of cells expressing p16 (a controller of senescence), it was demonstrated that DOX-induced cardiac dysfunction is associated with increased myocardial senescence, and the elimination of p16-expressing senescent cells rescues cardiac function (Demaria et al., 2017). Suggesting that mitochondrial dysfunction and increased oxidative stress are key inducers of senescence in CVD, including AIC, are studies demonstrating that mitochondria-targeted overexpression of catalase increases longevity and reduces age-related cardiac pathologies (Dai et al., 2017), and heart-specific overexpression of catalase increases resistance to DOX-induced cardiotoxicity (Kang et al., 1996). Although it is not yet known how senescent cells contribute to the pathophysiology of AIC, data from other senescence-associated CVD models provide clues (Zhu et al., 2015; Anderson et al., 2019; Correia-Melo et al., 2019; Lewis-McDougall et al., 2019; Walaszczyk et al., 2019; Dookun et al., 2020). During natural aging, cardiomyocytes obtain a senescent phenotype, induced by telomere-localized DNA damage which can occur independently of proliferative capacity and telomere shortening (Anderson et al., 2019). Senescent cardiomyocytes have characteristics that promote myocardial remodeling as they are hypertrophic and express a profibrotic and pro-hypertrophic SASP (Anderson et al., 2019; Walaszczyk et al., 2019).

Senescence within the cardiac fibroblast population has also been linked to myocardial remodeling, contributing to increased fibrosis. Cardiac fibroblasts are crucial to the formation and maintenance of the extracellular matrix (ECM) in the cardiac microenvironment and are necessary for the proper contractility and function of cardiomyocytes (DeLeon-Pennell et al., 2020). However in response to injury or stress, fibroblasts are activated and undergo myofibroblast differentiation, and produce the intracellular contractile protein alpha-smooth muscle actin (α-SMA), therefore increasing ECM production and becoming pro-inflammatory (Baum and Duffy, 2011). Interestingly, in pressure overload and cardiac ischemia-reperfusion models, cardiac myofibroblasts undergo cellular senescence and secrete known SASP-related proteins including TGF-β, IL6, and MCP-2 which contribute to ECM production and fibrosis (Meyer et al., 2016; Dookun et al., 2020). Moreover, senescent myofibroblasts demonstrate other typical senescence-related changes including increased reactive oxygen species generation and resistance to apoptosis (Gibb et al., 2020). Fibrotic lesions are found in the hearts of patients treated with anthracyclines, and studies have demonstrated that rats chronically exposed to DOX develop cardiac fibrosis because of increased collagen production and fibroblast survival (Levick et al., 2019). Although, to the best of our knowledge, there have been no clear demonstrations that anthracyclines induce cardiac fibroblasts or myofibroblasts to senescence *in vivo*, DOX induces primary cardiac fibroblasts to senescence *in vitro* (Espitia-Corredor et al., 2022), and DOX is routinely used to induce fibroblasts from other organ systems to senescence (Kilic Eren et al., 2015; Bientinesi et al., 2022). Suggesting senescence contributes to fibrosis and remodeling, cardiac fibroblasts lacking expression of p53, a key controller of senescence, exposed to DOX demonstrated reduced cell cycle arrest, increased proliferation, displayed reduced migration, and attenuated expression of genes associated with dilated cardiomyopathy (Mancilla et al., 2020).

DOX can also impact the function of other myocardial-resident cells including endothelial cells and can induce coronary microcirculation dysfunction in small animal and pig models (Huang et al., 2010; Eckman et al., 2013; Galán-Arriola et al., 2021). *In vitro*, DOX induces endothelial cells to a senescent-like phenotype which includes dysregulation of vascular tone, increased endothelium permeability, arterial stiffness, impairment of angiogenesis and vascular repair, and a reduction in mitochondrial biogenesis (Wolf and Baynes, 2006; Jia et al., 2019). Endothelial cell senescence has been implicated in the development of CVDs including coronary artery disease, aortic aneurysm, and stenosis during aging (Childs et al., 2016; Roos et al., 2016; Balint et al., 2019), and therefore, it likely contributes to cardiac dysfunction following cancer therapy, as reviewed previously (Abdelgawad et al., 2022b).

## Senescence as a therapeutic target for anthracycline-induced cardiotoxicity

Although transgenic mouse models that facilitate the pharmacogenetic elimination of senescent cells have provided compelling evidence that senescence contributes to DOX-induced cardiac dysfunction, these models lack the potential for clinical translation. Fortunately, over the last 5 years, several small molecules have been identified that can selectively eliminate senescent cells. Senescent cells express pro-survival networks which promote survival and apoptosis resistance (Wang, 1995). Based on these observations, Zhu et al. demonstrated that compounds inhibiting these survival pathways induce Senescent but not proliferative cells to apoptosis (Zhu et al., 2015; Yosef et al., 2016). These compounds have been termed senolytics.

Recently, one such senolytic navitoclax (ABT-263), a Bcl-2 family protein inhibitor (Tse et al., 2008), was used to eliminate senescence in a model of DOX-induced cardiac dysfunction (Lérida-Viso et al., 2022). Mice treated with concomitant administration of DOX and navitoclax had fewer senescent cells in their myocardium and an increase in the expression of Sur2a, a marker of cardiac stress tolerance, which decreases in cardiac aging (Baker et al., 2016), and had been reduced by DOX treatment alone. Furthermore, although control DOX-treated mice demonstrated a significant decline in fractional shortening, a measurement of cardiac function, navitoclax-treated mice demonstrated no such decline and maintained a cardiac function comparable to non-DOX-treated mice throughout the study (4 weeks). Although Lérida-Viso et al. showed that navitoclax treatment reduced myocardial senescence, they did not investigate which senescent cell lineages were reduced by treatment. However, navitoclax has demonstrated *in vitro* senolytic activity in multiple myocardial-resident cell lineages, including cardiomyocytes and primary endothelial cells (Anderson et al., 2019; Abdelgawad et al., 2022a), and in the context of other disease models, senolytics have shown the potential to eliminate cell populations including cardiomyocytes, fibroblasts, endothelial cells, and even lymphocytes, all of which have roles in pathological myocardial remodeling (Childs et al., 2016; Dookun et al., 2020; Martin-Ruiz et al., 2020). Furthermore, in the context of aging and cardiac ischemia-reperfusion, navitoclax mediates the elimination of both cardiomyocyte and non-cardiomyocyte senescent populations and attenuates inflammation and myocardial remodeling, reducing both fibrosis and hypertrophy (Anderson et al., 2019; Walaszczyk et al., 2019; Dookun et al., 2020). It is therefore probable that navitoclax mediates protection from AIC *via* the elimination of multiple senescent cell types and their corresponding SASP.

**FIGURE 2:**
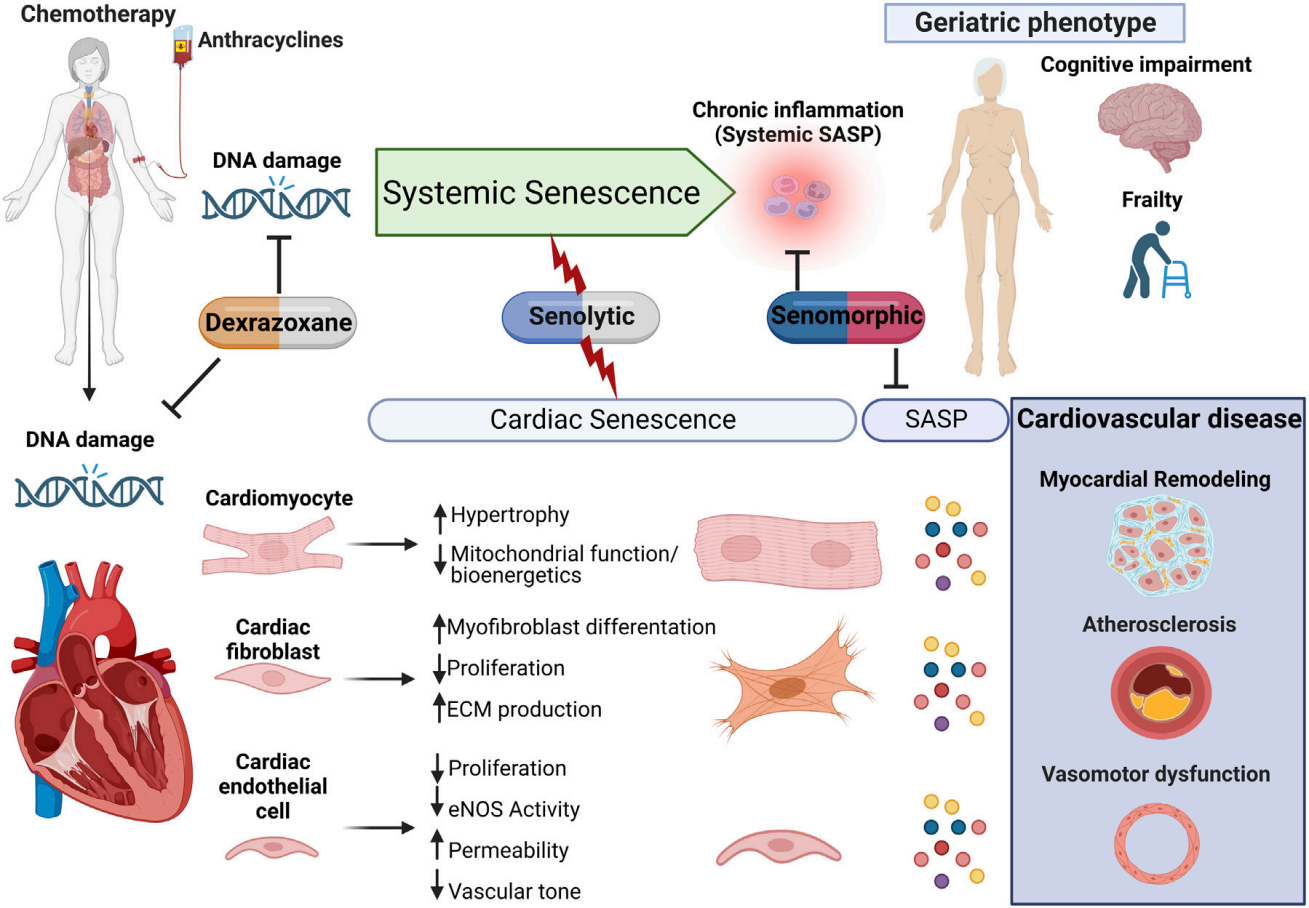
Proposed model for anthracycline-induced senescence in cardiotoxicity. We propose a model in which anthracyclines, through several interdependent mechanisms culminating in persistent DNA damage, induce systemic senescence and drive a geriatric phenotype. In the heart, senescence is induced in several cell populations which collectively contribute to age-related cardiovascular diseases, including myocardial remodeling, both directly and *via* expression of the senescence-associated secretory phenotype (SASP). This raises therapeutic opportunities through either elimination of senescent cells or modulation of the senescent phenotype for the management of anthracycline-induced cardiotoxicity and potentially less well-studied systemic toxicity.

It is important to remember that senolytics eliminate senescent cells *via* the induction of apoptosis, and while the heart has some regenerative potential, this is limited and reduces with age (Bergmann et al., 2015; Richardson et al., 2015; Richardson, 2016). As such, questions remain regarding the safety of senolytics since myocardial cell death, particularly in the cardiomyocyte population, is usually associated with cardiomyopathy and progression to heart failure (Hausenloy and Yellon, 2013). Additional studies are, therefore, required to evaluate the heart’s potential to maintain function, following the induction of senescent cell apoptosis in the longer term. Fortunately, alternative strategies that target senescence without cell loss have been identified. Senomorphic agents modulate the senescent cell phenotype by interfering with senescence-related signaling pathways and SASP expression, without induction of apoptosis (Niedernhofer and Robbins, 2018). The senomorphic drug rapamycin can reduce senescent cell burden and attenuate myocardial remodeling in a mouse model of senescence and accelerated aging (Correia-Melo et al., 2019). Moreover, metformin (which reduces cardiovascular mortality and cardiovascular events in patients suffering from coronary heart disease (Han et al., 2019) and has a cardioprotective effect in small animal models of AIC (Zilinyi et al., 2018)) has senomorphic activity suppressing senescence and SASP expression in cell populations including fibroblasts (Moiseeva et al., 2013), endothelial cells (Arunachalam et al., 2014), and smooth muscle cells (Tai et al., 2021). Although the precise mechanisms by which metformin elicits its cardioprotective effect are not fully understood, it is possible that interference with the senescent phenotype may contribute. There is currently great interest in the small-molecule telomerase activator TA-65, which has been shown to induce telomerase activity in different tissues within mouse and human primary cells (Fauce et al., 2008; de Jesus et al., 2011; Richardson et al., 2018; Hoffmann et al., 2021), and protect against senescence *via* telomerase-dependent elongation of short telomeres and against mitochondrial dysfunction and oxidative stress. In mice, TA-65 treatment attenuated pathological myocardial remodeling and improved cardiac function post-myocardial infarction (Ale-Agha et al., 2021). Interestingly, while the cardioprotective mechanisms of dexrazoxane are not fully understood, dexrazoxane has been shown to prevent DOX-induced DNA damage *via* depleting both topoisomerase II isoforms (Deng et al., 2014). As such, attenuation of senescence, as a result of DNA protection, could contribute to the cardioprotective effects of this clinically used therapy (Figure 2).

## Beyond anthracyclines

Chemotoxicity and associated life-threatening cardiac effects are currently largely associated with and studied in the context of anthracyclines. Due to improvements in cancer survivorship, it is now becoming evident that delayed and chronic cardiotoxicity is also caused by more recently developed second- and third-generation chemotherapies (Florescu et al., 2013; Michel et al., 2019). Although evidence remains sparse and is limited to studies of the murine skin, studies have shown that newer chemotherapeutics including paclitaxel, temozolomide, and cisplatin can induce senescence (Demaria et al., 2017). As such, novel strategies targeting senescence may have wider applications to delay, prevent, or even reverse the deleterious effects of chemotherapy more broadly.
